# Reply to B.O. Anderson et al

**DOI:** 10.1200/JGO.2016.006262

**Published:** 2016-08-31

**Authors:** Linus T. Chuang, Nagi S. El Saghir, Sarah Temin, Jonathan S. Berek

**Affiliations:** **Linus T. Chuang**, Icahn School of Medicine at Mt Sinai, New York, NY; **Nagi S. El Saghir**, American University of Beirut Medical Center, Beirut, Lebanon; **Sarah Temin**, American Society of Clinical Oncology, Alexandria, VA; **Jonathan S. Berek**, Stanford Comprehensive Cancer Institute, Stanford, CA

We appreciate the insight and comments of Anderson and Duggan^1^ in their correspondence “Resource-Stratified Guidelines for Cancer Management: Correction and Commentary” in response to the ASCO publication of “Management and Care of Women with Cervical Cancer: American Society of Clinical Oncology Resource-Stratified Clinical Practice Guideline.”^2^ We welcome Anderson and Duggan’s^1^ appreciation of the new ASCO resource-stratified guidelines and gratefully acknowledge the pioneering role of the Breast Health Global Initiative (BHGI) in resource-stratified guideline development. BHGI’s leadership has enriched the sources of guidance for clinicians, policy makers, and others. ASCO was fortunate and pleased to partner with and participate in BHGI as described by Anderson and Duggan,^1^ including the participation by individual ASCO members in BHGI. We thank them for kindly providing the historic note on ASCO’s participation, to which we add ASCO’s role in the BHGI Alliance 2012/2013 and 2010/2011.^3^

Anderson and Duggan^1^ state that ASCO Expert Panel authors “have incorrectly attributed their resource-stratification methodology to the World Health Organization (WHO) rather than to BHGI, and that they have also incorrectly cited the peer-review publications describing the concepts of resource stratification that they have applied to the management of invasive cervical cancer.”^1^ They state that this concept should properly be attributed to BHGI, which was the first to develop, test, and validate the concept of a four-tiered resource stratification.^1^

We acknowledge that the ASCO Expert Panel on Cervical Cancer resource-stratified guidelines did mention that “in developing resource-stratified guidelines, ASCO has adopted its framework from the four-tier approach (basic, limited, enhanced, and maximal, summarized in Table 1 and Appendix Table A2) developed by WHO and applied by the Breast Health Global Initiative and made modifications to that framework based on Disease Control Priorities 3 (DCP3) and uses an evidence-based approach to inform guideline recommendations.”^2(p2)^ Although we agree with Anderson and Duggan^1^ that this particular ASCO guideline's reference is incorrectly cited, and that the four-tier resource stratification is attributable to BHGI, we believe that the both approaches are interrelated and that a brief summary of them is helpful for readers, investigators, and health care authorities.

The 2002 WHO publication, referenced by both BHGI^5^ and ASCO,^2^ described three resource scenarios under the heading of “national cancer control activities based on resource realities.”^4(p24)^ WHO described resource scenarios A, B, and C (low-resource, middle-resource, and high-resource scenarios) to facilitate establishment of national cancer control plans that ensure the most efficient use of existing resources in the control of cancer.

WHO defined countries with a low level of resources (scenario A), where resources for chronic disease are completely absent or limited, and where cancer is not one of the primary health problems, but for those older than 15 years it can be one of the leading causes of death. Health care services are often delivered by informal means, and alternative medicine is a major component of care. Infrastructure and human resources for cancer prevention or control are nonexistent or limited in quantity, quality, and accessibility. They recommend establishing a basis for prevention of cancer and other chronic diseases by combatting smoking and reducing dependence on westernized diet, educating public and health care workers about the early warning signs of cancer and other diseases, as well as establishing a basis for pain relief and palliative care for individuals with advanced disease.^4^

WHO defined medium level of resources (scenario B) for countries often considered middle-income, where cancer is usually one of the leading causes of disease and mortality. They have high exposure to risk factors, especially tobacco, diet, infectious agents, and carcinogens in the workplace, and limited infrastructure and human resources for cancer control. At this level, WHO recommended primary prevention and early detection, such as tobacco control, reduction of alcohol use, and promotion of healthy diet and physical exercise; attention to carcinogens in the workplace and to infectious agents such as human papilloma virus; and promotion of the warning signs for the common cancers. Cancer treatment should focus on cancers that are curable, and clinical trials should be encouraged to evaluate relatively low-cost approaches that eventually can be provided to all patients irrespective of their socioeconomic condition. More sophisticated approaches, such as radiotherapy and chemotherapy, should be introduced in specialized centers. Major efforts should be made to achieve the highest coverage for pain relief and palliative care, using low-cost drugs (oral morphine) and other interventions.^4^

High level of resources (scenario C) is that of industrialized countries with a relatively high level of resources for health care, where life expectancy is more than 70 years and cancer is a major cause of death for men and women, with many existing elements of a cancer control program that might not be well integrated into a comprehensive national system. Prevention and early detection programs need to be improved, and serious deficiencies with respect to providing easy access to pain relief and palliative care services often need to be addressed. The WHO document outlines recommendations for minimum essential actions by national cancer control programs for countries with different levels of resources.^6,7^

On the other hand, in 2006 BHGI described four scenarios, termed a four-tier resource stratification framework (framework: basic, limited, enhanced, and maximal resource levels), in which cancer management strategies can be prioritized within the context of available health care resources.^5^ BHGI described service availability as a function of levels of resources of the country. Countries at the basic level need to have and develop fundamental services to take care of patients with cancer (example: surgery). Countries that are at the limited resource level should have services that could produce major improvement in outcome, with limited costs (example: tamoxifen for breast cancer). Patients in countries with enhanced levels of resources have choices of treatment modalities that have limited improvement in outcome, and in countries with maximal resources all options are available, even with minimal improvements.^5^

BHGI offered descriptions of available infrastructures and human resources and published comprehensive guideline recommendations for better use of resources—that is, to do the best the countries can with the resources that they have, for all aspects of breast cancer control, including prevention, awareness, early detection, diagnosis, radiology, pathology, surgery, chemotherapy, hormonal therapy, radiation therapy and supportive and palliative care, health care systems, and resources, as well as the important aspects of implementation of guidelines in subsequent summit meetings and BHGI and individual publications.^6,8-15^ As noted by Anderson and Duggan,^1^ ASCO, as well as WHO, Union for International Cancer Control, and other major organizations, have been supportive and collaborated with BHGI in its various summit meetings.^3^

Although BHGI’s work focused on women with breast cancer, their model is applicable to other cancers, as the ASCO Resource-Stratified Guidelines Advisory Group and others have demonstrated. Adapting BHGI’s model, ASCO began to undertake resource-stratified guideline development in 2013, leveraging ASCO’s guideline development expertise and international membership, on another globally high-incidence cancer: cervical cancer.

The initial leaders of ASCO’s Resource-Stratified Guidelines Advisory Group included BHGI authors Nagi El-Saghir and Eduardo Cazap. ASCO is fortunate that its cervical cancer–related Resource-Stratified Clinical Practice Guideline Expert Panels and larger Consensus Ratings Panels' members are from or have extensive experience in basic and limited (indeed, all four) resource settings and/or participate in or were leaders in BHGI, DCP3, and the Asian Oncology Summit guidance. We note the inclusion of health economists with expertise in these settings in our Resource-Stratified Guidelines initiative.

We acknowledge BHGI and BHGI’s volunteers’ enormous contributions. BHGI’s four-tier resource stratification for breast cancer was extremely helpful as we undertook guideline development. In some cases, we customized the framework to fit cervical cancer. Detailed reviews of other ASCO methodologies used are available in the guideline’s data and methodology supplements, including a description of formal consensus, systematic review, literature search terms, Appraisal of Guidelines for Research and Development II (AGREE II) results, and more.

In the short term, *Journal of Oncology Practice* summary states “ASCO has adopted its framework from the four-tier approach (basic, limited, enhanced, and maximal) developed by the Breast Health Global Initiative and modifications to that framework based on the DCP3 and used an evidence-based approach to inform guideline recommendations”^17^^(p3)^ and cites Anderson et al.^5^ In future ASCO Resource-Stratified Guideline publications, ASCO will be pleased to cite BHGI’s 2006 publications^5,18-20^ and may publish an explanatory note or paper on ASCO methodology.

A minor point: the letter from Anderson and Duggan^1^ refers to ASCO Consensus Panels; the ASCO nomenclature is ASCO Expert Panels. When ASCO recruits a larger group of experts to participate in the formal consensus process in addition to the Expert Panel, the ASCO term is Consensus Ratings Panel.^21^

The authors are in complete agreement with the last paragraph of Anderson and Duggan.^1^ International collaborations, especially with the participation of target implementers, are crucial to move toward globally affecting outcomes for people with cancer and those caring for them.
